# Correlations between circulating adipokines and hepatocellular carcinoma: a Systematic Review and meta-analysis

**DOI:** 10.3389/fendo.2025.1548924

**Published:** 2025-06-18

**Authors:** Yani Ke, Yuyan Pan, Xueru Huang, Xing Bai, Xiaojuan Liu, Mingsi Zhang, Tao Jiang, Guangji Zhang

**Affiliations:** ^1^ School of Basic Medical Sciences, Zhejiang Chinese Medical University, Hangzhou, Zhejiang, China; ^2^ Key Laboratory of Blood-stasis-toxin Syndrome of Zhejiang Province, Hangzhou, Zhejiang, China; ^3^ Traditional Chinese Medicine “Preventing Disease” Wisdom Health Project Research Center of Zhejiang, Hangzhou, Zhejiang, China; ^4^ The First Affiliated Hospital of Zhejiang Chinese Medical University (Zhejiang Provincial Hospital of Traditional Chinese Medicine), Hangzhou, Zhejiang, China; ^5^ School of Sport, Exercise and Health Sciences, Epinal Way, Loughborough University, Loughborough, Leicestershire, United Kingdom

**Keywords:** HCC, hepatocellular carcinoma, adipokine, adiponectin, leptin, meta-analysis

## Abstract

**Background:**

Hepatocellular carcinoma (HCC) is among the most common malignant tumors, characterized by high incidence and mortality rates. The role of adipokines in liver diseases is increasingly recognized and involves multiple contributing factors. Therefore, we summarized the relationship between circulating adipokines and HCC to guide directions for future research.

**Methods:**

Six databases were searched, and all data were presented as standardized mean difference (SMD) or weighted mean difference (WMD). Sensitivity analysis and meta-regression were also performed. Diagnostic meta-analysis results were primarily presented using receiver operating characteristic (ROC) curves.

**Results:**

A total of 41 articles were included in this meta-analysis. HCC patients had significantly higher levels of circulating adiponectin, leptin, visfatin, and resistin compared to the controls (SMD = 1.6, 95% CI: 0.65-2.56; SMD = 2.45, 95% CI: 1.59-3.31; SMD = 2.49, 95% CI: 1.32-3.65; SMD = 4.17, 95% CI: 3.17-5.17, respectively). Conversely, circulating irisin levels in HCC patients were significantly lower than those in the control group (WMD = -1.16, 95% CI: -1.55, -0.77). Subgroup analysis identified possible sources of heterogeneity, whereas meta-regression confirmed that only the presence or absence of viral hepatitis was the source of high heterogeneity among leptin-related studies. Additionally, the meta-analysis results of diagnostic studies show that circulating visfatin demonstrates good diagnostic value for HCC, which may be helpful for clinical practice.

**Conclusion:**

There is a significant association between circulating adipokines and HCC, and the presence of viral hepatitis is an influencing factor. Most adipokines are differentially expressed in HCC patients, and some may serve as biomarkers for early diagnosis or prognostic assessment.

**Systematic review registration:**

https://www.crd.york.ac.uk/PROSPERO/, identifier CRD42023492972.

## Introduction

Hepatocellular carcinoma (HCC) is one of the most common cancers worldwide, accounting for about 90% of all primary liver cancer cases (1, 2). It ranks sixth in incidence worldwide and is the second leading cause of cancer-related deaths (3, 4). The development of HCC is considered a multi-step pathological process that progresses from chronic hepatitis to cirrhosis (5), with various etiologies and triggers, including aflatoxin-contaminated food, excessive alcohol consumption, and obesity (6). In clinical management, the prognosis and treatment of HCC depend on tumor staging: early-stage HCC can be treated with local ablation, surgical resection, and liver transplantation, intermediate-stage HCC is typically treated with chemoembolization, while advanced-stage HCC is managed with systemic chemotherapy, tyrosine kinase inhibitors (TKIs), and immunotherapy (7). Due to the lack of specific early screening and diagnostic biomarkers, HCC is often diagnosed at an advanced stage, resulting in missed opportunities for optimal treatment or a high risk of recurrence after resection (8). Therefore, a deeper understanding of the mechanisms underlying HCC and the identification of effective biomarkers for early diagnosis and prognosis are crucial for clinical practice.

Adipokines are peptides produced by adipose tissue. They have autocrine, paracrine, and endocrine functions (9, 10), and regulate various physiological systems. Because the liver plays a central role in systemic lipid and glucose metabolism, hepatocytes are highly susceptible to ectopic lipid accumulation (11). Many studies have shown that adipokines can regulate liver function: changes in adipokines occur during adipose tissue expansion and contribute to the development of nonalcoholic steatohepatitis (NASH) and may lead to cirrhosis, ultimately evolving into HCC (12, 13). However, many current studies on the expression of circulating adipokines in patients with HCC report inconsistent or conflicting results. Moreover, fatty infiltration has been identified in HCC with high differentiation (14) and a steatohepatitic subtype of HCC has also been defined (15), but whether adipokines affect it and to what extent adipokines affect it are still unclear. A deeper understanding of the relationship between adipokines and HCC may support their use as non-invasive biomarkers for early screening and diagnosis, and provide real-time detection tools for HCC treatment and prognosis. Therefore, we conducted a systematic review and meta-analysis of common circulating adipokines in HCC to explore their clinical and scientific value from multiple perspectives.

## Method

### Literature search

This study adheres to the PRISMA guidelines, as detailed in **Additional File 1**. The registered protocol is available on the PROSPERO website (registration number: CRD42023492972) and detailed in **Additional File 2**. Two members of the team searched a total of six databases, including PubMed, Embase, Cochrane Library, CNKI, Wanfang, and CBM. The search was conducted up to December 18, 2024, in both English and Chinese. The search strategy combined MeSH terms with free words, and the main keywords included “HCC”, “hepatocellular carcinoma”, “hepatic cancer”, “adipokine”, “adiponectin”, “leptin”, “visfatin”, “resistin”, “irisin”, “chemerin” and “apelin”. The selection of adipokines was based on articles on liver diseases (9, 10, 16). The specific retrieval strategies for different databases are presented in **Additional File 3**. Additionally, the references of the included articles were also screened manually. For articles with incomplete information or inaccessible full text, we attempted to contact the corresponding authors via email.

### Study selection

The search results from each database were independently browsed and filtered by two members. Any disagreements were resolved by a third reviewer in accordance with the protocol. The specific rules are as follows:

Studies were included if they met all of the following criteria: (1) The research subjects are patients with HCC; (2) The control group includes healthy individuals without HCC and other serious diseases (such as other cancers, severe diseases of the heart, brain, and other organs, etc.), aged ≥ 18; (3) The result must contain one or more circulating adipokines (including adiponectin, apelin, chemerin, leptin, visfatin, resistance and irisin); (4) cohort study or case-control study.

The study was excluded if any of the following conditions were met: (1) The control group includes patients with other liver diseases (such as cirrhosis, after liver transplantation, etc.); (2) Non-circulating adipokine results, or adipokine indicators outside our research protocol; (3) Studies performed in the same cohort; (4) Articles with missing key data and inability to contact the original author; (5) Case reports, review articles, or duplicate publications.

### Data extraction and quality assessment

Data extraction and quality evaluation were also independently completed by two members, and any uncertainties or disputes were handed over to a third person for judgment. The extracted data included publication year, country or region of the study population, the Newcastle-Ottawa Scale (NOS) score, diagnostic method or basis for HCC, basic information of the case group and control group (number of people, age, gender, etc.), measured values and detection methods of circulating adipokines, comorbidities in the case group, diagnostic analysis results (including sensitivity, specificity, cutoff, and area under receiver operating characteristic (ROC) curve), etc. Among them, NOS score (17, 18) was used to evaluate the methodological quality of the included studies, which includes the assessments of selection, comparability, and exposure.

### Statistical analysis

We used various software to analyze data, including Stata12 (StataCorp, in Texas, USA) for basic meta-analysis, and Meta DiSc 1.4 (Clinical Biostatistics Unit, in Madrid, Spain) for diagnostic meta-analysis. The data included in the study were ultimately presented in the form of mean ± standard deviation (SD) and summarized into an Excel 2016 (Microsoft, in Washington, USA). Among them, normally distributed data can be directly used, while non-normally distributed data needed to be evaluated and converted into standard form (18–20) (specific website is https://www.math.hkbu.edu.hk/~tongt/papers/median2mean.html). Considering the inconsistency among units in various studies, most data were presented in the form of standardized mean difference (SMD) and Confidence Intervals (CI). For meta-analysis using the same unit (like μg/ml, ng/l, etc.), weighted mean difference (WMD) and CI were adopted. Heterogeneity was assessed using the I^2^ test and Cochrane’s Q test, presented in a Galbraith test. The fixed-effect model was suitable when heterogeneity was not significant (I^2^<50%) (21, 22). When heterogeneity is significant (I^2^>50%), a random-effect model was used. To further clarify the possible sources of heterogeneity, subgroup analysis and meta-regression would also be applied. The specific application was determined by the number of studies included. Sensitivity analysis was conducted by sequentially excluding individual studies to assess the robustness of the results, which can be used to judge the stability of the summarized research results. Publication bias is also an important aspect that cannot be ignored, so this study used Egger’s test (23) to measure it. If there was a significant publication bias, we would further evaluate the reliability of the results through trim-and-filling method (24). The trim-and-filling method can test the impact of publication bias by iteratively estimating and adding potentially missing studies, ensuring that meta-analysis results are not severely distorted by potential bias, thereby enhancing the reliability and robustness of the research. In addition, the meta-analysis results of diagnostic analysis mainly include the summary results of sensitivity, specificity, positive likelihood ratio (PLR), negative likelihood ratio (NLR), diagnostic odds ratio (DOR), and summary Receiver Operating Characteristic (sROC). Different studies have different threshold effects, which has a significant impact on the results of diagnostic meta-analysis. If the threshold effect is not significant (p>0.05), the heterogeneity differences among studies are not significant, and the results are somewhat convincing. If the threshold effect is significant (p<0.05), the sources of heterogeneity among studies need to be explored. In addition, the diagnostic value of expected indicators is determined by Area Under the Curve (AUC): AUC ≤ 0.5: poor accuracy; 0.5 < AUC ≤ 0.7: moderate accuracy; 0.7 < AUC ≤ 0.9: good accuracy; AUC = 1: perfect accuracy (25).

## Results

### Study selection

**Figure 1:**
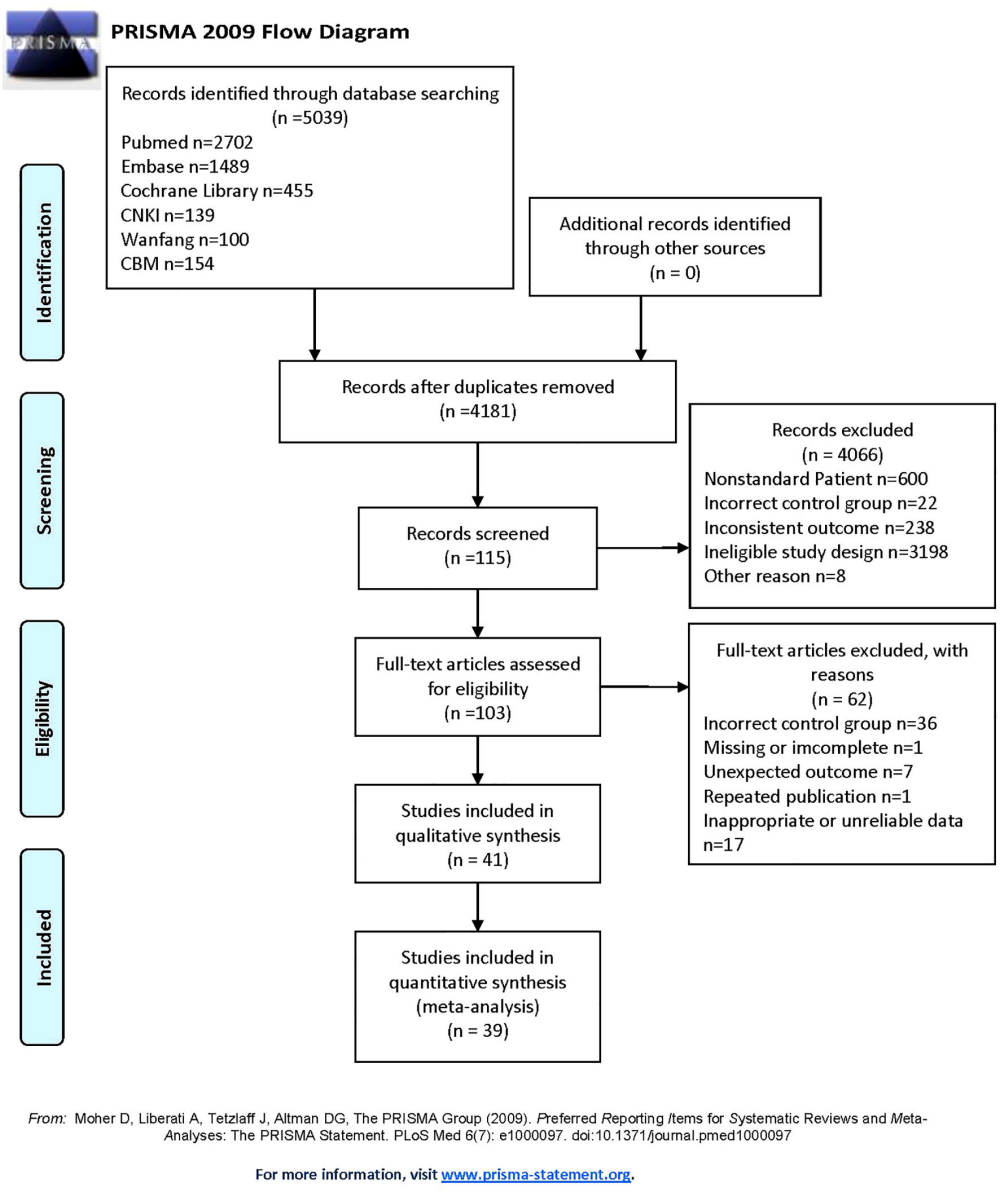
Flowchart of study inclusions and exclusions: PRISMA 2009 flow diagram.

After searching six databases, a total of 5039 articles was found. Duplicate literature was removed, and 4181 articles still needed to be screened. According to the inclusion and exclusion criteria set earlier, 41 studies remained in qualitative synthesis. Among them, 5 studies (26–30) were suitable for data conversion and relevant data were obtained through the methods described earlier (18–20). Furthermore, since one study mentions circulating apelin and one mentions chemerin, only 39 studies were ultimately included in the quantitative analysis. These studies covered various regions in Asia, Africa, and Europe, with a total of 2778 HCC patients and 2637 controls in this meta-analysis. Our study mainly covered five indicators: circulating adiponectin, leptin, visfatin, resistin and irisin. The publication period of the included studies is from 2003 to 2024, with a long time span. The specific information of the included studies can be seen in **Table 1** (26–66), and the overall screening process is shown in **Figure 1**.

**Table 1:** Baseline characteristics of studies included.

No.	Author	Year	Country/ Region	No. of Patients		Sex (M/F)		Age		Age-matching	Combined with Viral hepatitis	Detection Method	Adipokine
				HCC group	Control group	HCC group	Control group	HCC group	Control group				
1	Abdelhamed (31)	2023	Egypt	37	20	28/9	/	64.11 ± 8.09	/	/	All	ELISA	Visfatin
2	Abdelwahab (32)	2020	Egypt	30	15	/	/	/	/	/	/	ELISA	Leptin
3	Abouzied (26)	2017	Saudi Arabia	25	25	18/7	23/2	57.72 ± 4.75	29.15 ± 8.57	p<0.05	None	ELISA	Leptin
				25		16/9		59.82 ± 6.01					
4	Aleksandrova (33)	2014	Germany	125	250	85/40	171/79	60.1 ± 6.6	60.1 ± 6.6	p>0.05	Not all	ELISA	Adiponectin, Leptin
5	Ataseven (34)	2006	Turkey	22	25	15/7	11/14	59.82 ± 8.77	37.12 ± 7.57	p<0.05	Not all	ELISA	Leptin
6	Çavuş (35)	2020	Turkey	60	20	45/15	14/6	59.2 ± 10.2	53.4 ± 8.2	p<0.05	Not all	ELISA	Leptin
7	Chen (36)	2012	Taiwan	65	165	47/18	112/53	58.83 ± 12.93	47.67 ± 10.08	p<0.05	Not all	RIA	Adiponectin
8	Chen (37)	2020	China	112	112	55/57	59/53	53.48 ± 12.59	50.37 ± 12.46	p>0.05	/	ELISA	Visfatin
9	Cheng (38)	2010	China	42	30	/	/	67.5 ± 10.7	63.3 ± 10.4	p>0.05	Not all	ELISA	Leptin
10	Costantini (27)	2013	Italy	26	20	18/8	9/11	/	/	p>0.05	All	ELISA	Leptin
11	Dai (39)	2010	China	82	102	71/11	80/22	/	/	p>0.05	All	ELISA	Leptin
12	Dai (40)	2015	China	48	48	38/10	/	/	52.5 ± 6.5	/	Not all	RIA	Adiponectin
13	Duan (41)	2012	China	34	17	34/0	17/0	/	/	/	/	ELISA	Leptin
14	Ebrahim (42)	2012	Egypt	120	50	74/46	28/22	50 ± 10	48 ± 7	p>0.05	Not all	ELISA	Adiponectin
15	Effenberger (28)	2023	Austria	20	11	16/4	7/4	46 ± 4.83	25.48(4)	p<0.05	/	ELISA	Apelin
16	Elsayed (43)	2015	Egypt	100	50	85/15	24/26	52.3 ± 6.2	51 ± 7	p>0.05	All	ELISA	Resistin
17	Gaggini (44)	2017	Italy	18	18	/	11/7	54.9 ± 6.5	67.2 ± 14.4	p<0.05	Not all	ELISA	Irisin
18	Gu (45)	2012	China	80	40	45/35	/	54.2 ± 10.7	/	/	/	ELISA	Leptin
19	Guo (46)	2012	China	64	60	/	30/30	58.1 ± 4.9	56.5 ± 3.4	p<0.05	/	ELISA	Adiponectin
20	Hou (47)	2006	China	146	30	101/45	15/15	46.2 ± 10.7	38.5 ± 9.8	p<0.05	Not all	ELISA	Leptin
21	Karam (48)	2020	Egypt	100	50	20/30	75/25	55.0 ± 1.5	59.8 ± 5.1	p<0.05	Not all	ELISA	Leptin
22	Khattab (49)	2012	Egypt	147	320	114/33	201/119	43.9 ± 4.7	42.9 ± 10.3	p>0.05	/	Luminex xMAP technology	Adiponectin
23	Kotani (50)	2009	Japan	59	334	33/26	195/139	63.5 ± 7.3	62.7 ± 6.9	p>0.05	Not all	ELISA	Adiponectin
24	Li (51)	2012	China	27	30	20/7	20/10	/	/	/	/	RIA	Leptin
25	Liang (52)	2017	China	60	40	37/23	25/15	52.73 ± 11.34	51.26 ± 10.8	p>0.05	/	ELISA	Visfatin
26	Liu (53)	2005	China	2	30	2/0	18/12	59.5 ± 12.02	39.43 ± 12.33	p>0.05	All	ELISA	Leptin
27	Liu (54)	2009	Taiwan	120	116	100/20	67/49	50.7 ± 13.8	53.8 ± 10.5	p>0.05	All	ELISA	Adiponectin
28	Liu (55)	2012	China	48	30	34/14	21/9	53 (31–80)	52(43-81)	p<0.05	Not all	ELISA	Resistin
				56		48/8		57(41-74)					
29	Luo (56)	2008	China	30	30	21/9	18/12	48.55 ± 10.5	50.0 ± 15.0	p>0.05	/	RIA	Leptin
30	Pazgan (57)	2018	Poland	70	20	/	/	60.0 ± 12.1	55.6 ± 6.9	p<0.05	Not all	ELISA	Visfatin
31	Pazgan (58)	2020	Poland	69	20	54/15	10/10	59.0 ± 9.0	40.6 ± 5.5	p<0.05	Not all	ELISA	Irisin
32	Pazgan (59)	2024	Poland	13	10	/	/	63 (42–75)	44.3(21–67)	p<0.05	/	ELISA	Chemerin
				32				58.5(46–77)					
33	Piao (60)	2006	China	10	20	5/5	10/10	/	/	p>0.05	All	ELISA	Adiponectin
34	Sadik (29)	2012	Egypt	69	21	43/26	13/8	/	55.7 ± 3.54	p>0.05	All	ELISA	Adiponectin, Leptin
35	Tao (61)	2008	China	64	59	/	/	51 ± 11	48 ± 9	p>0.05	/	ELISA	Leptin
36	Ti (62)	2010	China	190	100	141/49	39/61	57.3 (44–67)	42.3(39-47)	/	/	RIA	Leptin
37	Tsai (30)	2017	Taiwan	36	92	26/10	68/24	62.0 ± 10.9	51.8 ± 5.9	p<0.05	Not all	ELISA	Visfatin
				39		21/18		69.4 ± 9.7		p<0.05	Not all		
38	Wang (63)	2003	Taiwan	31	25	/	/	65 ± 2	65 ± 2	p>0.05	Not all	RIA	Leptin
39	Xu (64)	2016	China	38	50	/	25/25	/	36.33 ± 7.37	/	All	ELISA	Adiponectin
40	Ye (65)	2008	China	70	30	50/20	13/17	45.8(23-72)	43.7(22-65)	/	/	RIA	Leptin
41	Zhang (66)	2019	China	117	102	94/23	82/20	54.7 ± 11.1	53.6 ± 10.2	p>0.05	Not all	ELISA	Irisin

HCC, Hepatocellular Carcinoma; ELISA, enzyme-linked-immunosorbent serologic assay; RIA, radioimmunoassay.

### Quality assessment

Two members (XJ L and MS Z) conducted quality evaluation separately, and any disputes were decided by a third person (GJ Z). The NOS scores of the 41 studies (26–66) included vary, with the highest score being 7 and the lowest 5. The average NOS score is 5.71 (shown in **Table 2**), indicating that most articles adopted a reasonable experimental design and a clear experimental process.

**Table 2:** NOS score of included studies.

No.	Author	Year	Country/Region	Selection				Comparability	Exposure			Total	Average	Adipokine
				Adequate definition	Representativeness	Selection of controls	Definition of controls		Ascertainment of exposure	Same method	Nonresponse rate			
1	Abdelhamed (31)	2023	Egypt	1	1	0	1	0	1	1	0	5	5.71	Visfatin
2	Abdelwahab (32)	2020	Egypt	1	1	0	1	0	1	1	0	5		Leptin
3	Abouzied (26)	2017	Saudi Arabia	1	1	1	1	1	1	1	0	7		Leptin
4	Aleksandrova (33)	2014	Germany	1	1	1	1	1	1	1	0	7		Adiponectin, Leptin
5	Ataseven (34)	2006	Turkey	1	1	0	1	0	1	1	0	5		Leptin
6	Çavuş (35)	2020	Turkey	1	1	0	1	0	1	1	0	5		Leptin
7	Chen (36)	2012	Taiwan	1	1	1	1	1	1	1	0	7		Adiponectin
8	Chen (37)	2020	China	1	1	1	1	1	1	1	0	7		Visfatin
9	Cheng (38)	2010	China	1	1	1	1	0	1	1	0	6		Leptin
10	Costantini (27)	2013	Italy	1	1	0	1	0	1	1	0	5		Leptin
11	Dai (39)	2010	China	1	1	1	1	1	1	1	0	7		Leptin
12	Dai (40)	2015	China	1	1	1	1	0	1	1	0	6		Adiponectin
13	Duan (41)	2012	China	1	1	0	1	0	1	1	0	5		Leptin
14	Ebrahim (42)	2012	Egypt	1	1	0	1	1	1	1	0	6		Adiponectin
15	Effenberger (28)	2023	Austria	1	1	0	1	0	1	1	0	5		Apelin
16	Elsayed (43)	2015	Egypt	1	1	1	1	0	1	1	0	6		Resistin
17	Gaggini (44)	2017	Italy	1	1	0	1	0	1	1	0	5		Irisin
18	Gu (45)	2012	China	1	1	0	1	0	1	1	0	5		Leptin
19	Guo (46)	2012	China	1	1	0	1	0	1	1	0	5		Adiponectin
20	Hou (47)	2006	China	1	1	0	1	0	1	1	0	5		Leptin
21	Karam (48)	2020	Egypt	1	1	1	1	0	1	1	0	6		Leptin
22	Khattab (49)	2012	Egypt	1	1	1	1	0	1	1	0	6		Adiponectin
23	Kotani (50)	2009	Japan	1	1	0	1	1	1	1	0	6		Adiponectin
24	Li (51)	2012	China	1	1	0	1	0	1	1	0	5		Leptin
25	Liang (52)	2017	China	1	1	1	1	1	1	1	0	7		Visfatin
26	Liu (53)	2005	China	1	1	0	1	1	1	1	0	6		Leptin
27	Liu (54)	2009	Taiwan	1	1	1	1	0	1	1	0	6		Adiponectin
28	Liu (55)	2012	China	1	1	1	1	1	1	1	0	7		Resistin
29	Luo (56)	2008	China	1	1	0	1	1	1	1	0	6		Leptin
30	Pazgan (57)	2018	Poland	1	1	0	1	0	1	1	0	5		Visfatin
31	Pazgan (58)	2020	Poland	1	1	0	1	0	1	1	0	5		Irisin
32	Pazgan (59)	2024	Poland	1	1	0	1	0	1	1	0	5		Chemerin
33	Piao (60)	2006	China	1	1	0	1	1	1	1	0	6		Adiponectin
34	Sadik (29)	2012	Egypt	1	1	0	1	1	1	1	0	6		Adiponectin, leptin
35	Tao (61)	2008	China	1	1	0	1	1	1	1	0	6		Leptin
36	Ti (62)	2010	China	1	1	0	1	0	1	1	0	5		Leptin
37	Tsai (30)	2017	Taiwan	1	1	0	1	0	1	1	0	5		Visfatin
38	Wang (63)	2003	Taiwan	1	1	0	1	0	1	1	0	5		Leptin
39	Xu (64)	2016	China	1	1	0	1	0	1	1	0	5		Adiponectin
40	Ye (65)	2008	China	1	1	0	1	0	1	1	0	5		Leptin
41	Zhang (66)	2019	China	1	1	1	1	1	1	1	0	7		Irisin

### Association between adiponectin and HCC

**Figure 2:**
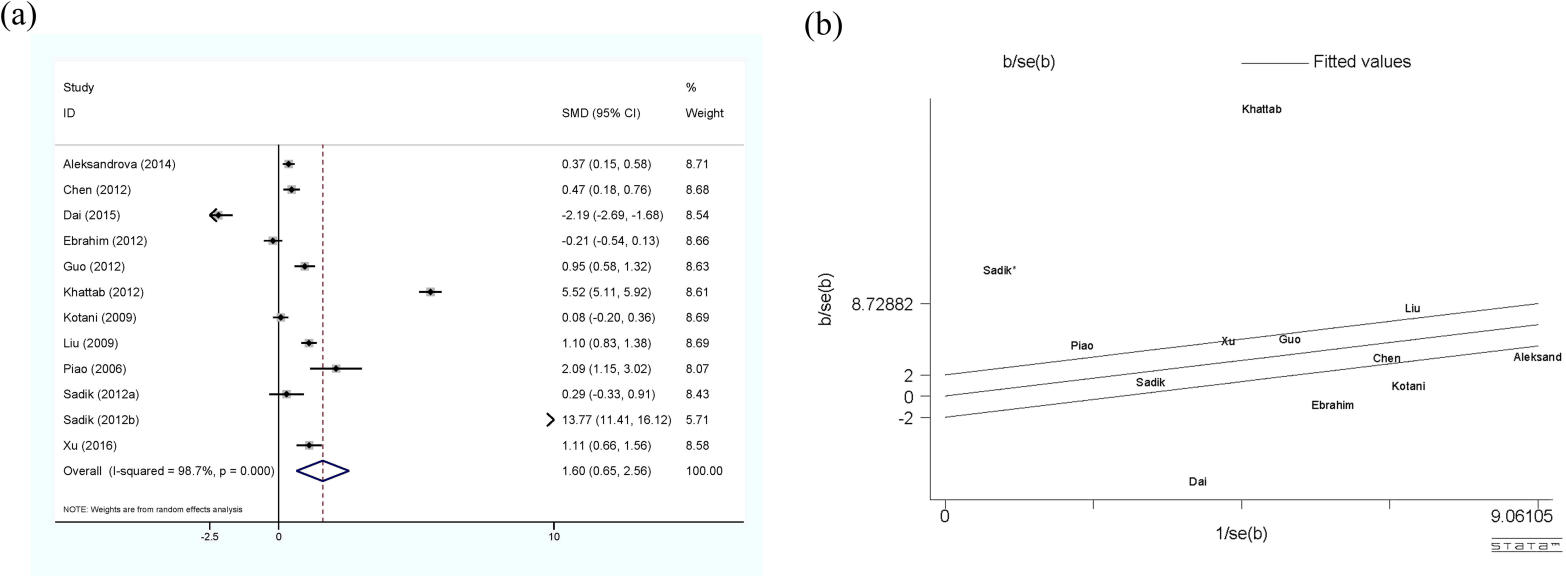
**(a)** Forest plot of circulating adiponectin levels between HCC and the control group: SMD = 1.65 (0.65, 2.56); **(b)** Galbraith Test result of circulating adiponectin levels between HCC and the control group: half of the studies included are out of the fitted values. SMD, standardized mean difference; CI, Confidence Intervals.

The meta-analysis of adiponectin and HCC is shown in **Figure 2**, using the random-effect model. Although there was significant heterogeneity (I^2^ = 98.7%, p<0.01) among the 11 studies included (12 sets of data), the results clearly showed that circulating adiponectin levels in HCC patients were significantly higher than those in the control group, with SMD 1.6 (0.65-2.56). Meanwhile, the Galbr result also demonstrates high heterogeneity among these studies, as most of them are not within a reasonable range. Therefore, subgroup analysis and meta-regression were performed to identify potential sources of heterogeneity.

All subgroup analysis results are presented in **Table 3**, by stratifying the combined data according to area (East Asia and others), detection method (ELISA and others), age-match (p>0.05 and p<0.05), viral hepatitis (all and not all) and sex. Only one study (49) provides circulating adiponectin levels in HCC patients of different Tumor Node Metastasis (TNM), so this could not be taken as the basis of subgroup analysis. No obvious sources of heterogeneity were found as a result. However, the influence of the area where the HCC group came from and whether HCC was accompanied by viral hepatitis cannot be ignored. There was no significant difference in circulating adiponectin levels between HCC patients from East Asia and the control group (SMD=0.49, 95% CI: -0.23, 1.21), while HCC patients from other regions had significantly higher circulating adiponectin levels than the control group (SMD=3.62, 95% CI: 1.23, 6.01). The result of HCC patients all combined with viral hepatitis (29, 54, 60, 64) showed higher circulating adiponectin levels than the control group (SMD=2.91, 95% CI: 1.46, 4.35), but HCC patients some combined with viral hepatitis (33, 36, 40, 42, 50) had no significant difference in circulating adiponectin levels compared with the control group (SMD=-0.26, 95% CI: -0.91, 0.39). With regard to the studies including HCC patients some combined with viral hepatitis (33, 36, 40, 42, 50), the viral hepatitis rate (HBsAg (+)/anti-HCV Ab (+)/anti-HDV Ab (+)) of each study was as follows: 32%, 90.8%, 85.4%, 76.7% and no specific data available. Thus, to clarify the specific sources of heterogeneity, meta-regression was further performed, with results presented in **Table 4**. Unfortunately, none of the selected factors were potential sources of heterogeneity (p>0.05), which deserves further attention in the future.

**Table 3:** Subgroup analysis of circulating adiponectin level in patients with HCC and the control group.

Subgroup	Data sets	Model	SMD (95%CI)	P	I^2^
Area					
East Asia	7	Random	0.49 [-0.23, 1.21]	<0.00001	96.2%
Others	5	Random	3.62 [1.23, 6.01]	<0.00001	99.4%
detection method					
ELISA	9	Random	1.29 [0.66, 1.92]	<0.00001	95.8%
Others	3	Random	1.27 [-2.74, 5.28]	<0.00001	99.7%
age-match					
age-match (p>0.05)	8	Random	2.48 [1.14, 3.82]	<0.00001	99%
age-not-match (p<0.05)	2	Random	0.7 [0.23, 1.16]	0.048	74.5%
viral hepatitis					
All	5	Random	2.91 [1.46, 4.35]	<0.00001	96.7%
not all	5	Random	-0.26 [-0.91, 0.39]	<0.00001	95.7%
sex*					
male/female	5	Random	-0.06 [-1.05, 0.93]	<0.00001	90.6%

SMD, standardized mean difference; CI, Confidence Intervals.

*Horizontal comparison between HCC patients with different characteristics.

**Table 4:** Meta-regression of circulating adiponectin levels and HCC.

Covariates	Data sets	Coefficient	Standard error	t	P	95%CI
Univariate meta-regression analysis						
Area	12	25.735	57.519	1.45	0.177	[0.177, 3743.938]
detection method	12	0.449	1.254	-0.29	0.78	[0.001, 226.637]
combined viral hepatitis	10	0.023	0.057	-1.51	0.169	[0.000, 7.356]
age-match	10	0.127	0.436	-0.6	0.565	[0.000, 352.603]

CI, confidence interval.

### Association between leptin and HCC

**Figure 3:**
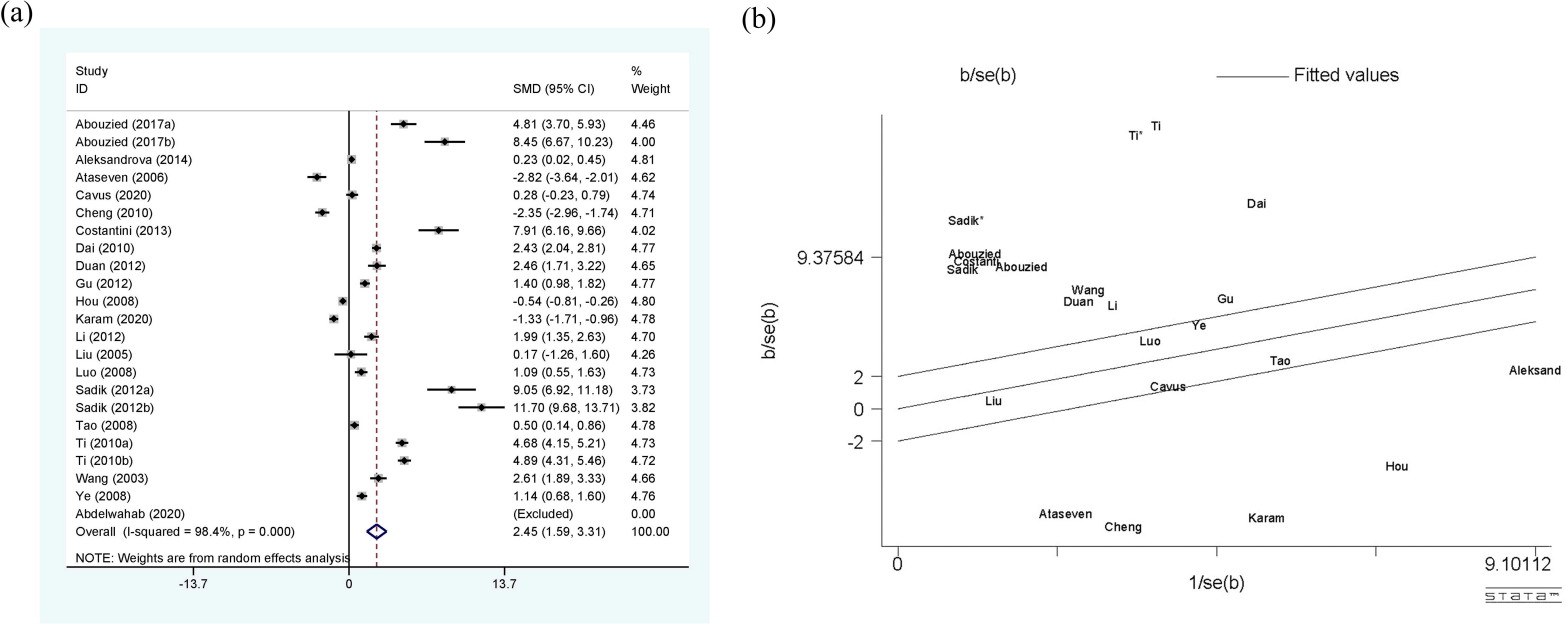
**(a)** Forest plot of circulating leptin levels between HCC and the control group: SMD = 2.45 (1.59, 3.31); **(b)** Galbraith Test result of circulating leptin levels between HCC and the control group: more than half of the studies included are out of the fitted values. SMD, standardized mean difference; CI, Confidence Intervals.

**Figure 3** shows a comprehensive meta-analysis of circulating leptin and HCC, which includes 20 studies and 23 sets of data. Due to the presence of high heterogeneity (I^2^ = 98.4%, p<0.01), the random-effect model was selected. The results confirm that the expression of circulating leptin levels in HCC group is significantly higher than that in the control group, with SMD 2.45 (1.59-3.31). The Galbr plot shows that most studies are not within a reasonable range, indicating the presence of high heterogeneity. Therefore, subgroup analysis and meta-regression became methods for exploring the sources of heterogeneity.

As shown in **Table 5**, the perspectives of subgroup analysis include area (East Asia, Middle East and others), detection method (ELISA and others), age-match (p>0.05 and p<0.05), viral hepatitis (all and not all), TNM (stage I, II and stage III, IV), and sex. Among them, age-match and whether viral hepatitis is present may be sources of heterogeneity, but the results are not very significant. For age-matched studies, the circulating leptin levels in HCC patients were significantly higher than those in the control group (SMD=2.96, 95% CI: 1.72, 4.19). On the contrary, for studies performed without age matching, there was no significant difference in circulating leptin levels between the two groups (SMD=1.24, 95% CI: -0.30, 2.78). In terms of the studies where all case participants had viral hepatitis (27, 29, 39, 53) compared to those partially accompanied with viral hepatitis (33–35, 48, 63), HCC patients all with viral hepatitis showed significantly higher levels of circulating leptin than the control group (SMD=6.17, 95% CI: 2.46, 9.88), while the difference between the two groups with only some cases of concomitant viral hepatitis was not significant (SMD=-0.20, 95% CI: -1.40, 1.00). With regard to the studies including HCC patients some combined with viral hepatitis (33–35, 48, 63), the viral hepatitis rate (HBsAg (+)/anti-HCV Ab (+)/anti-HDV Ab (+)) of each study was as follows: 32% (HBsAg (+)/anti-HCV Ab (+)), 59.1% (HBsAg (+)), 66.7% (HBsAg (+)/anti-HCV Ab (+)), 85% (anti-HCV Ab (+)) and 61.3% (HBsAg (+)/anti-HCV Ab (+)). In addition, we also compared the levels of circulating leptin between male and female patients and patients with different TNM stages (47, 48, 65). It is obvious that the circulating leptin levels in female patients are significantly higher than those in male patients, with SMD -2.71 (-4.20, -1.21). In order to clarify whether the above factors were possible sources of heterogeneity, we also conducted a univariate meta-regression (see **Table 6**) and verified that presence of viral hepatitis is indeed one of the sources of heterogeneity (p=0.025), which may have important implications for clinical practice.

**Table 5:** Subgroup analysis of circulating leptin level in patients with HCC and the control group.

Subgroup	Data sets	Model	SMD (95%CI)	P	I^2^
Area					
East Asia	13	Random	1.58 [0.55, 2.61]	<0.00001	98.3%
Middle East	8	Random	4.14 [1.46, 6.82]	<0.00001	98.7%
Europe	2	Random	4.02 [-3.50, 11.54]	<0.00001	98.6%
detection method					
ELISA	15	Random	1.32 [0.45, 2.20]	<0.00001	97.9%
Others	8	Random	4.38 [2.83, 5.94]	<0.00001	97.7%
age-match					
age-match (p>0.05)	10	Random	2.96 [1.72, 4.19]	<0.00001	98%
age-not-match (p<0.05)	6	Random	1.24 [-0.30, 2.78]	<0.00001	98%
viral hepatitis					
All	5	Random	6.17 [2.46, 9.88]	<0.00001	97.4%
not all	5	Random	-0.20 [-1.40, 1.00]	<0.00001	97.3%
TNM					
I、II	3	Random	-0.24 [-1.34, 0.86]	<0.00001	94.2%
III、IV	3	Random	-0.61 [-1.52, 0.29]	<0.00001	90.3%
TNM*					
I、II/III、IV	3	Random	0.85 [0.32, 1.38]	0.057	65%
sex*					
male/female	5	Random	-2.71 [-4.20, -1.21]	<0.00001	96.5%

SMD, standardized mean difference; CI, Confidence Intervals.

*Horizontal comparison between HCC patients with different characteristics.

**Table 6:** Meta-regression of circulating leptin levels and HCC.

Covariates	Data sets	Coefficient	Standard error	t	P	95%CI
Univariate meta-regression analysis						
Area	22	5.471	6.562	1.42	0.172	[0.448, 66.787]
detection method	22	20.94	32.97	1.93	0.068	[0.785, 558.864]
combined viral hepatitis	10	573.387	1324.098	2.75	0.025	[2.791, 117796.9]
age-match	16	6.192	14.207	0.79	0.44	[0.045, 849.166]

CI, Confidence Intervals.

### Association between visfatin and HCC

**Figure 4:**
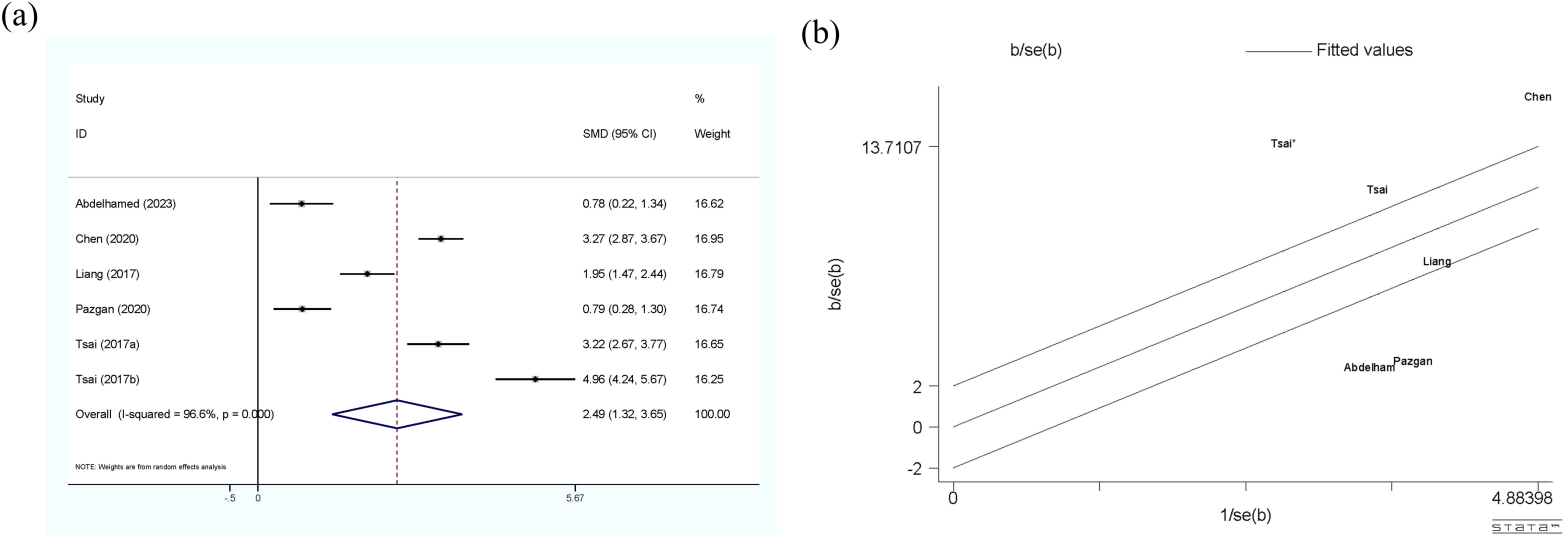
**(a)** Forest plot of circulating visfatin levels between HCC and the control group: SMD=2.49 (1.32, 3.65); **(b)** Galbraith Test result of circulating visfatin levels between HCC and the control group: more than half of the studies included are out of the fitted values. SMD, standardized mean difference; CI, Confidence Intervals.

Five studies (six sets of data) explored the relationship between circulating visfatin levels and HCC, and their meta-analysis results are shown in **Figure 4**. According to the results, HCC patients showed significantly higher circulating visfatin levels than the control group (SMD=2.49, 95% CI: 1.32, 3.65), using a random-effect model. High heterogeneity is reflected in the I^2^ = 96.6%, p<0.01, and Galbr plot. Most studies are not within a reasonable range, indicating significant heterogeneity. Due to the limited number of studies included, only subgroup analysis was applied (see **Table 7**). Unfortunately, sources of heterogeneity were not found.

**Table 7:** Subgroup analysis of circulating leptin level in patients with HCC and the control group.

Subgroup	Data sets	Model	SMD (95%CI)	P	I^2^
Area					
East Asia	4	Random	3.33 [2.28, 4.37]	<0.00001	93.8%
Others	2	Random	0.79 [0.41, 1.17]	0.974	0%
age-match					
age-match (p>0.05)	2	Random	2.62 [1.33, 3.91]	<0.00001	94%
age-not-match (p<0.05)	3	Random	2.98 [0.65, 5.31]	<0.00001	97.9%
Unit					
the same unit	4	Random	2.72 [1.12, 4.31]	<0.00001	97%
different units	2	Random	2.03 [-0.41, 4.47]	<0.00001	98%

SMD, standardized mean difference; CI, Confidence Intervals.

**Figure 5:**
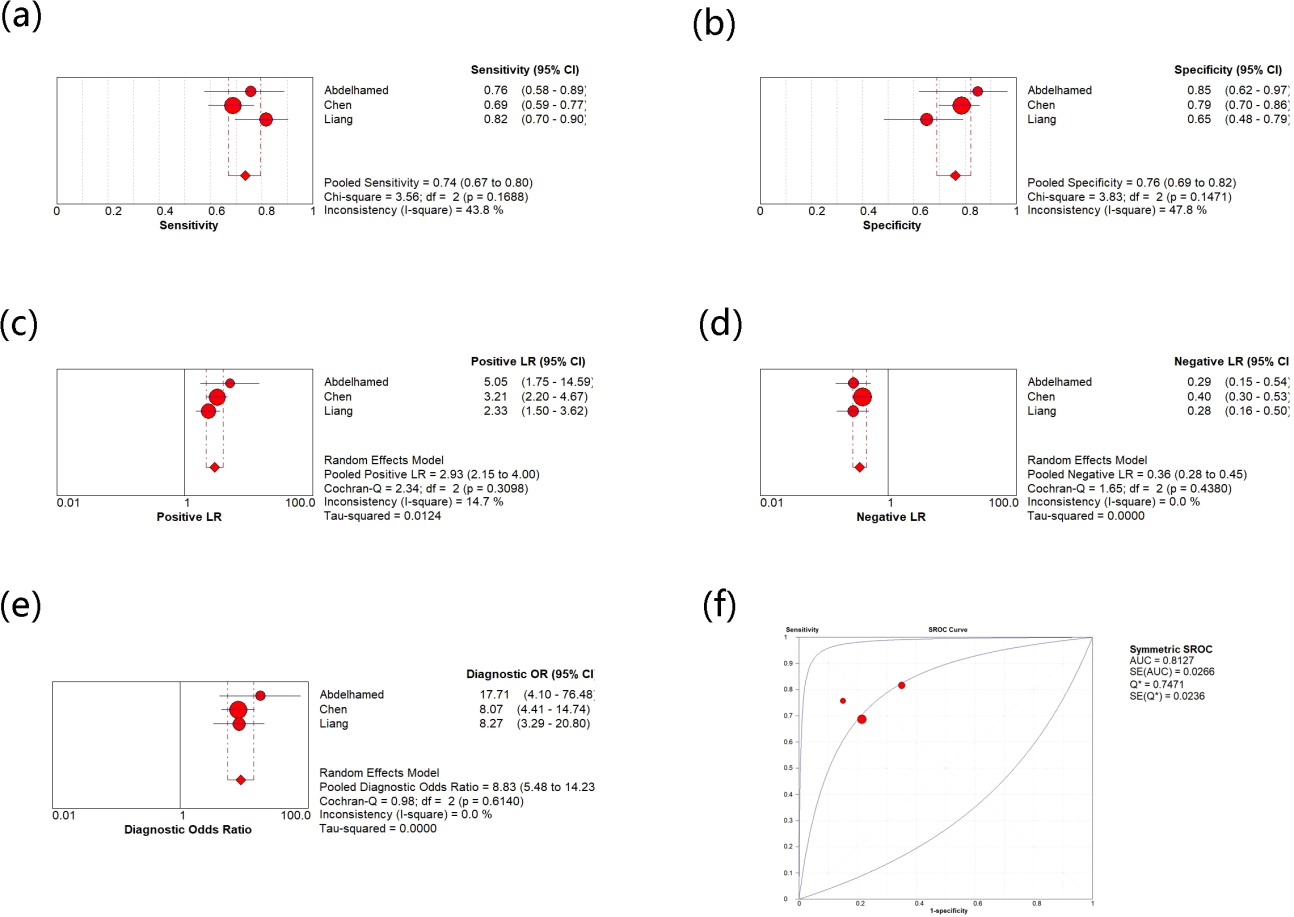
**(a)** Meta-analysis of sensitivity for circulating vasfatin in diagnosing HCC; **(b)** Meta-analysis of specificity for circulating vasfatin in diagnosing HCC; **(c)** Meta-analysis of PLR for circulating vasfatin in diagnosing HCC; **(d)** Meta-analysis of NLR for circulating vasfatin in diagnosing HCC; **(e)** Meta-analysis of DOR for circulating vasfatin in diagnosing HCC; **(f)** ROC curve of circulating visfatin in diagnosing HCC. CI, Confidence Intervals; PLR, Positive Likelihood Ratio; NLR, Negative Likelihood Ratio; DOR, Diagnostic Odds Ratio; ROC, Receiver Operating Characteristic; AUC, Area Under the Curve.

Moreover, there are three studies on the use of circulating visfatin as a diagnostic tool for HCC, so the relevant meta-analysis was also performed (see **Figure 5**). The comprehensive analysis shows that the sensitivity and specificity values are 0.74 (0.67, 0.80) and 0.76 (0.69, 0.82), and the I^2^ values for sensitivity and specificity are 43.8% and 47.8%, respectively, indicating low heterogeneity among the three studies. The pooled results of PLR, NLR, and DOR are 2.93 (2.15, 4.0), 0.36 (0.28, 0.45), 8.83 (5.48, 14.23), respectively, and their heterogeneity is not significant. Moreover, the comprehensive AUC curve was plotted and the result showed an AUC of 0.8127, indicating the good diagnostic value of using circulating visfatin to diagnose HCC. There was no significant threshold effect among the three diagnostic studies included, further demonstrating the reliability of all the results.

### Association between resistin and HCC

**Figure 6:**
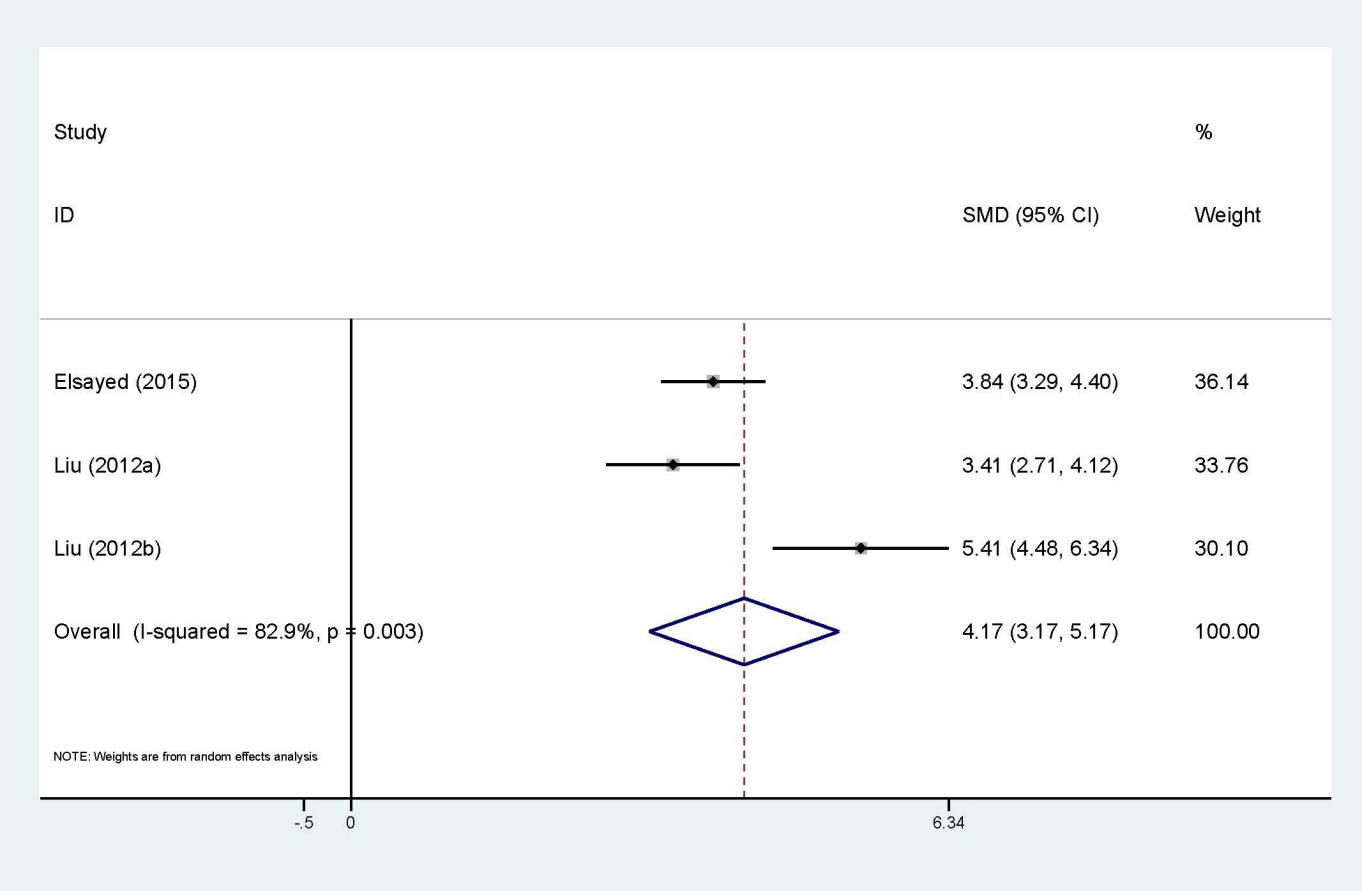
Forest plot of circulating resistin levels between HCC and the control group: SMD= 4.17 (3.17, 5.17). SMD, standardized mean difference; CI, Confidence Intervals.

Association between circulating resistin and HCC is presented in **Figure 6**, with 2 studies (3 sets of data). Although only two studies were included, high heterogeneity (I^2^ = 82.9%, p<0.01) still exists, which explains why a random-effect model was chosen. The results confirm that the expression of circulating resistin levels is significantly higher in HCC patients than in the control group (SMD=4.17, 95% CI: 3.17, 5.17). The number of studies included made it impossible to explore heterogeneity.

### Association between irisin and HCC

**Figure 7:**
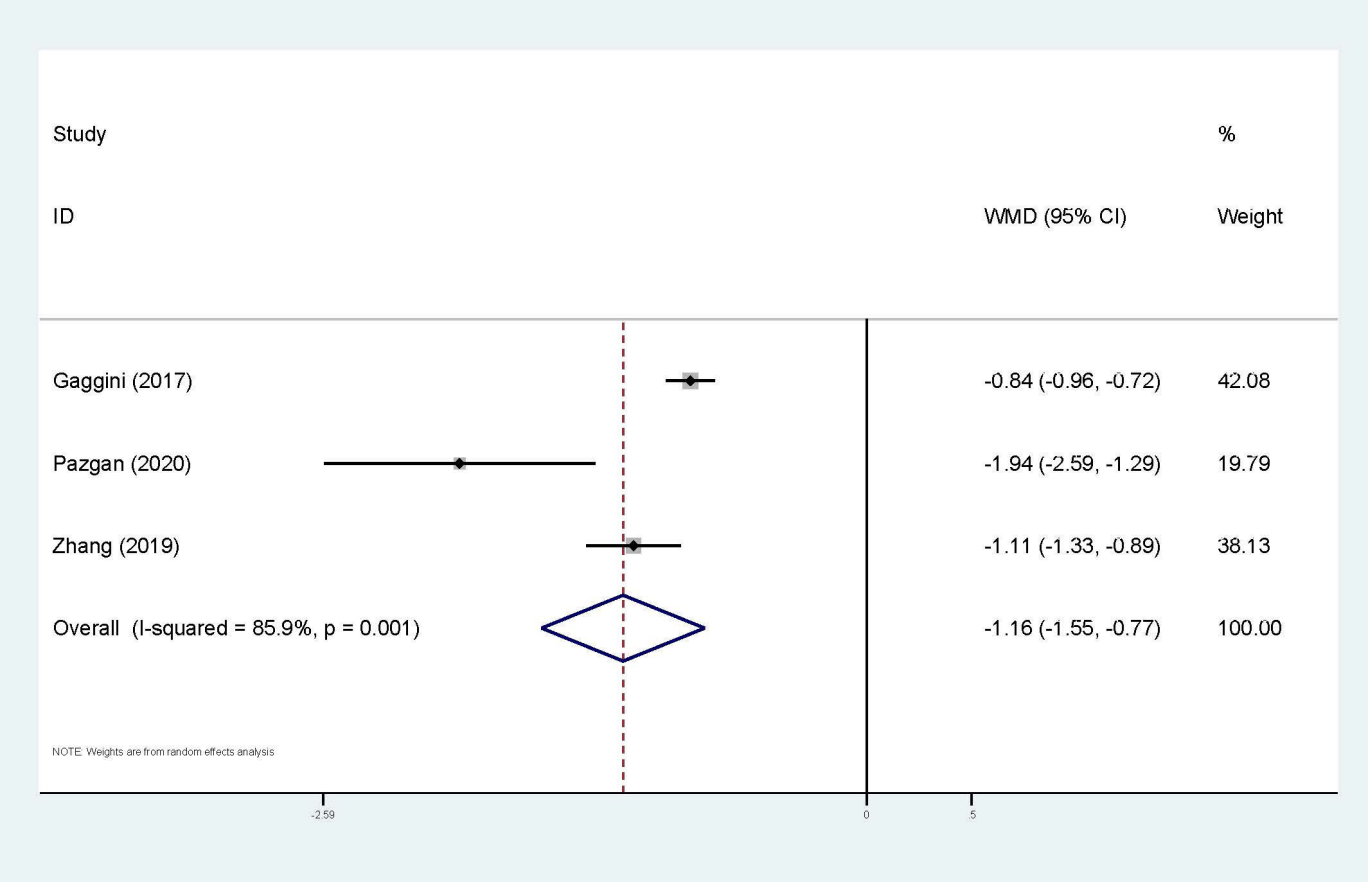
Forest plot of circulating irisin levels between HCC and the control group: WMD=-1.16 (-1.55, -0.77). WMD, weighted mean difference; CI, Confidence Intervals.

Three studies were applied to the meta-analysis of circulating irisin levels and HCC (**Figure 7**). Heterogeneity cannot be ignored (I^2^ = 86%, p<0.01), so the random-effect model has been applied. The results also showed that HCC patients had significantly lower levels of circulating irisin compared to the control group (WMD=-1.16, 95% CI: -1.55, -0.77). Exploration of heterogeneity was not conducted.

### Association between other adipokines and HCC

There is only one study on circulating apelin and HCC, and the result shows that circulating apelin levels in HCC patients are significantly higher than those in the control group (28). Furthermore, only one study was included on circulating chemerin and HCC (59), which suggests that HCC patients have higher circulating chemerin levels than the control group.

### Sensitivity analysis

**Figure 8:**
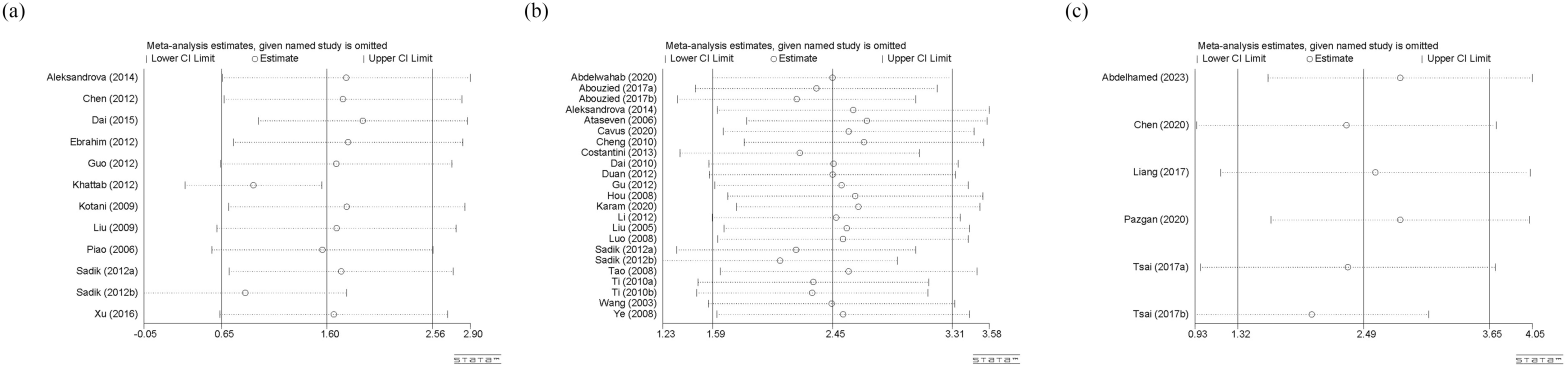
**(a)** Sensitivity analysis of circulating adiponectin levels; **(b)** Sensitivity analysis of circulating leptin levels; **(c)** Sensitivity analysis of circulating visfatin levels. CI, Confidence Intervals.

Sensitivity analysis was only conducted for circulating adiponectin, leptin, and visfatin (**Figure 8**). The stability of various meta-analysis results was observed by excluding each single study. If a study was excluded and the result completely deviated from the original scope, it indicates that the stability of the meta-analysis is poor. In our study, the sensitivity analysis results for any type of adipokines are reasonable and stable.

### Publication bias

**Figure 9:**
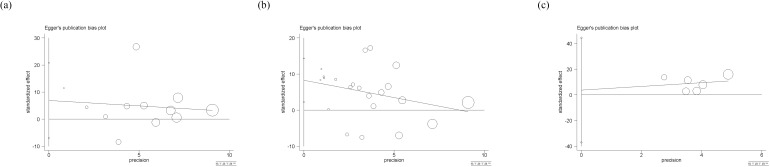
**(a)** Eggar’s test of circulating adiponectin levels; **(b)** Eggar’s test of circulating leptin levels; **(c)** Eggar’s test of circulating visfatin levels. The different sizes of symbols represent the weight of each study.

Egger’s test was used to evaluate publication bias, and the P-value was an important reference (**Figure 9**). Among them, there is a significant publication bias (p<0.05) for studies on circulating leptin and HCC, so we used the trim-and-filling method for further judgement. The result showed that no trimming was performed and no change happened, even with the addition of several articles. Besides, there is no significant publication bias (p>0.05) among studies on circulating adiponectin and visfatin, indicating that the results are representative.

## Discussion

The potential causes of liver cirrhosis are primarily alcoholism, chronic infections with hepatitis B virus (HBV) and hepatitis C virus (HCV), and NASH (67). Long-term cirrhosis can lead to repeated cycles of liver cell death and compensatory regeneration, creating an environment that promotes sustained cell growth and proliferation, which is highly beneficial for the occurrence and development of tumor cells (68). In addition, the pathogenesis of HCC related to obesity and non-alcoholic fatty liver disease (NAFLD) remains unclear, except for HCC caused by liver cirrhosis. Current research suggests that liver fat deformation combined with insulin resistance can promote adipose tissue inflammation, oxidative stress, and the occurrence of lipotoxins, thereby promoting liver cell carcinogenesis and facilitating liver cancer development (69–72). In recent years, there has been an increasing amount of research on HCC and adipokines, with varying focuses and results. Furthermore, research (73) has shown that the inflammatory biomarkers have been identified as key, especially for neuroendocrine neoplasms (NENs), highlighting the importance of early indicator monitoring for disease regulation.

Adiponectin is named after adipose tissue and can be produced by mature adipocytes or hepatocytes (9). Adiponectin mainly activates downstream pathways through two subtypes of receptors, namely R1 and R2 (16). Tontikodou et al. (74) found that patients combined with liver fibrosis and NAFLD had significantly lower levels of adiponectin than those without fibrosis. Jiang et al. (75) demonstrated that ethanol can damage the adiponectin R2- sirtuin 1 (SIRT1)- AMP-activated protein kinase (AMPK) signaling pathway, leading to the development of alcoholic fatty liver in rats. Leptin is a peptide hormone secreted by white adipose tissue (10). It can regulate satiety, energy expenditure, the immune system, angiogenesis, and carcinogenesis (76). It is highly expressed in many liver diseases and can promote the progression of fibrosis in patients with chronic hepatitis B infection (77, 78). Meanwhile, leptin can affect the tumor microenvironment and influence the invasion and distant metastasis of tumor cells in various ways, such as its interaction with cancer-related fibroblasts and the signaling of transforming growth factor beta (TGF-β) (79). Visfatin is a hormone derived from pre-B cell colony-enhancing factor, mainly expressed in bones, muscles, liver, and other tissues (80). Research has shown that the levels of visfatin are elevated in obese patients (81), and this cytokine can promote fibrotic activity (82). Resistin is a peptide secreted by adipocytes that promotes inflammation and insulin resistance (83, 84). Both animal experiments and clinical trials have found a correlation between resistin and obesity (11). In addition, resistin is positively correlated with inflammation and fibrosis in NAFLD (85, 86). Irisin is a novel glycopeptide hormone mainly produced and secreted by skeletal muscles (58), and can also be found in the liver, adipose tissue, and skin (87). It has a heat-generating effect on white adipose tissue during physical exercise, making it closely related to exercise (88). It can also regulate fat metabolism, thereby affecting liver lipid accumulation and determining the worsening prognosis of certain cancers (44, 89).

This meta-analysis explores the relationship between circulating adipokines and HCC, mainly including adiponectin, leptin, visfatin, resistin, irisin, chemerin, and apelin. After searching six databases, 5,039 articles were retrieved. Finally, a total of 41 studies were included, covering studies from Asia, Europe, and Africa. Among them, 11 articles contain circulating adiponectin levels, 20 articles involve circulating leptin, 5 articles are related to visfatin, 3 articles are resistin, 2 articles are irisin, 1 article is chemerin and 1 article is apelin. Relevant information was also extracted for subsequent subgroup analysis and meta-regression, including country, age, gender, testing methods, diagnostic criteria, comorbidities of HCC patients, detection methods, and units of circulating adipokines. Finally, it was found that HCC patients had significantly higher levels of circulating adiponectin, leptin, visfatin, and resistin compared to the control group, whereas circulating irisin levels were significantly lower in HCC patients. Due to the different number of studies included in each part, we only selected circulating adiponectin, leptin, and visfatin for subgroup analysis, and adiponectin and leptin for meta-regression.

After subgroup analysis, it was found that the expression levels of circulating adiponectin were different in HCC patients all combined with viral hepatitis and HCC patients some combined with viral hepatitis, compared with the control group. In addition, circulating adiponectin levels of HCC patients in other areas were significantly higher than those in the control group, while the circulating adiponectin levels of HCC patients in East Asia were no different from those in the control group. However, no possible sources of heterogeneity were found after meta-regression. Perhaps due to the limited number of studies included, it remains to be considered whether area and viral hepatitis are factors affecting the expression level of circulating adiponectin. In terms of circulating leptin, the results of subgroup analysis indicate that age matching, concomitant viral hepatitis, and gender all may contribute to a high degree of heterogeneity. Due to missing information in some studies, only the source of heterogeneity in the presence or absence of viral hepatitis was ultimately discovered through meta-regression. The subgroup analysis of visfatin did not find any sources of heterogeneity. Taking into account the heterogeneity exploration of these three circulating adipokines, we found that there are some differences in circulating adiponectin levels among patients in different areas. Even within the same area, there are significant differences in the levels of circulating adipokine, suggesting that the correlation between adipokine levels and geographic region may not be significant. In addition, there are differences in the degree of age matching among patients in different studies. It is generally accepted that liver metabolism gradually declines with age, so there may be a certain relationship between age and circulating adipokine. However, we only found that age may be a confounding variable reflected in a difference of circulating leptin between age matched and unmatched HCC subgroups, which may be due to limited relevant research on other adipokines and deserves further verification. The difference in hormone secretion between men and women may lead to the difference in the incidence rate or severity of HCC between the sexes. Therefore, we compared the level of circulating adipokine in male and female HCC patients and found that the level of circulating leptin was significantly different in male and female HCC patients. As before, this significant difference is only reflected in the study of circulating leptin, which may be related to the sensitivity of the indicator or the larger number of related studies. Viral hepatitis is currently the main factor leading to liver cancer (90). Viruses can promote the occurrence and development of liver cancer by affecting the lipid metabolism process of the liver (91). Therefore, the comorbidities of viral hepatitis in HCC patients can also affect the study outcomes and may be a key contributor to heterogeneity. Subgroup analyses of both circulating adiponectin and leptin showed that the presence or absence of viral hepatitis may lead to high heterogeneity. Meta-regression further suggests that the presence or absence of viral hepatitis has a significant impact on the expression of circulating adiponectin. Viral infection can cause persistent liver inflammation and immune mediated oxidative stress damage, further accelerating the transformation of inflammation to liver cancer, which may be the reason why it affects circulating adiponectin (92). A study suggests that there is an interaction between adipokines and HBV infection, HBV replication (including viral protein synthesis) (93). Moreover, we also explored the effects of detection methods, disease stage, and unit on heterogeneity, but no significant findings were observed, possibly due to the limited number of included studies. In fact, different disease stages and treatment of HCC patients may also affect lipid metabolism, thereby affecting the expression level of circulating adipokines. The focus of each study is different, and there is little information that can be summarized, which is worth further in-depth and comprehensive research in the future. It is worth mentioning that, unlike other circulating adipokines, irisin is expressed at lower levels in the circulatory system of HCC patients. The specific mechanism of irisin is not yet clear, and its similarities and differences with other circulating adipokines also need to be studied. Perhaps it can serve as a specific indicator for diagnosing and distinguishing liver diseases in the early stages.

In addition to routine meta-analysis, we also summarized the diagnostic analysis data for meta-analysis. Unfortunately, the diagnostic analysis data for many circulating adipokines are incomplete, so we only selected relevant studies on visfatin. The results showed no significant threshold effect, and circulating visfatin demonstrated high sensitivity, specificity, and diagnostic value for HCC. Based on these results, we believe that the level of circulating visfatin has good diagnostic significance for the diagnosis of HCC and deserves more experimental and clinical attention. We also conducted sensitivity analysis and analysis of publication bias to evaluate the reliability representativeness of the results. A potential publication bias was observed among studies on circulating leptin. The additional trim and filling method further confirms the reliability of the result. In general, there is a certain relationship between circulating adipokines and HCC: several types of circulating adipokines (adiponectin, leptin, visfatin, resistin) are expressed at higher levels in HCC patients, and whether HCC is combined with viral hepatitis can also affect the circulating adipokine levels of HCC patients. Additionally, circulating visfatin levels have good clinical value in diagnosing HCC.

### Limitations

This study covers the relationship between common circulating adipokines (adiponectin, leptin, visfatin, resistin and irisin) and HCC, but there are also some limitations. Firstly, the insufficient number of studies included has led to operational difficulties in subgroup analysis and meta-regression. Each study has its own focus and contains different data. It was difficult for us to extract the desired data from most studies, which also limited the scope of exploring heterogeneity. Secondly, although our research covers multiple countries and areas such as Asia, Europe, and Africa, it still mainly focuses on the Asian region and lacks research in areas such as the Americas. There are differences in the subgroup analysis of some indicators across different regions, but there are still some indicators that are not applicable. The area may be related to race or dietary habits, which can affect the incidence and severity of HCC (94) and is a point that needs to be further explored. Thirdly, there is only one indicator (circulating visfatin) for meta-analysis in diagnostic analysis. Because many of the included studies did not involve diagnostic studies or only provided ROC curve graphs, it is difficult for us to extract useful information from them. The meta-analysis of diagnostic studies summarized showed that circulating visfatin has a good diagnostic value for HCC. Whether this situation can be applied to other circulating adipokine indicators, or whether the combination of multiple circulating adipokine indicators can comprehensively clarify the incidence, disease severity, or prognosis of HCC, is an important direction for future research. Finally, in order to preliminarily elucidate the differences in circulating adipokine levels between HCC patients and healthy control groups, we conducted this study, and strictly selected healthy individuals as the control group (excluding other liver disease patients). However, cirrhosis could also be a confounder for adipokine levels, given potential alterations in nutritional status, liver synthetic function and the body’s inflammatory state. Perhaps more research can be conducted in the future to focus on the relationship between circulating adipokines and cirrhosis (95, 96). Furthermore, due to the limitation of article length, inconsistencies in objectives and multiple comorbidities with HCC, this article did not introduce a network meta-analysis and only explored the expression of these circulating adipokines in HCC patients to preliminarily clarify their clinical values. It is hoped that there will be opportunities to conduct a network meta-analysis in the future to further explore the comparison and collaboration of different adipokines in HCC and investigate their differences in the diagnosis and identification of liver diseases.

## Conclusion

This study summarizes the relationship between circulating adipokines and HCC, and some adipokines can provide a reference for the early diagnosis or prognostic evaluation of HCC. The expression levels of circulating adiponectin, leptin, visfatin, or resistin in HCC patients were significantly higher than those in the control group, while the circulating irisin level was significantly lower in HCC patients. All studies exhibited high heterogeneity, and subgroup analysis and meta-regression were conducted to identify the sources. Factors such as region and age-matching can be considered as possible sources of heterogeneity by subgroup analysis. However, due to the insufficient number of studies included, the meta-regression results demonstrated that the presence or absence of viral hepatitis is a source of high heterogeneity among studies on circulating leptin, which warrants further attention in future research. Additionally, the meta-analysis results of diagnostic studies suggest that circulating visfatin can serve as a good biomarker for diagnosing HCC. The results of the sensitivity analysis for all indicators are stable. By combining publication bias analysis and the trim-and-filling method, we have a clear understanding of the reliability of all the results in this study. However, due to the different focuses of each study, this meta-analysis still has many limitations, especially in exploring the sources of heterogeneity and the levels of circulating adipokines in various liver diseases. This requires further comprehensive and specific research to elucidate.

## Data Availability

The original contributions presented in the study are included in the article/**Supplementary Material**. Further inquiries can be directed to the corresponding authors.

## Supplementary material

Supplementary Data Sheet 1 PRISMA 2020 checklist.

Supplementary Data Sheet 2 PROSPERO: Number CRD42023492972.

Supplementary Data Sheet 3 Databases retrieval strategy.
