# Supplementary material for: Correlations between circulating adipokines and hepatocellular carcinoma: a Systematic Review and meta-analysis
Source: Front Endocrinol (Lausanne). 2025 Jun 18;16:1548924. doi: 10.3389/fendo.2025.1548924 (PMC12216435; doi:10.3389/fendo.2025.1548924)
Supplement: Supplementary Data Sheet 2 — PROSPERO: Number CRD42023492972. [file DataSheet2.pdf]

National Institute for Health Research

University of York
Centre for Reviews and Dissemination

Systematic review

A list of fields that can be edited in an update can be found here

1. * Review title.

Give the title of the review in English

The relationship between adipokine Levels and HCC: A Systematic Review and Meta-Analysis

2. Original language title.

For reviews in languages other than English, give the title in the original language. This will be displayed with the English language title.

3. * Anticipated or actual start date.

Give the date the systematic review started or is expected to start.

29/12/2023

4. * Anticipated completion date.

Give the date by which the review is expected to be completed.

29/03/2024

5. * Stage of review at time of this submission.

This field uses answers to initial screening questions. It cannot be edited until after registration.

Tick the boxes to show which review tasks have been started and which have been completed.

Update this field each time any amendments are made to a published record.

The review has not yet started: Yes

Review stage	Started	Completed
Preliminary searches	No	No
Piloting of the study selection process	No	No
Formal screening of search results against eligibility criteria	No	No
Data extraction	No	No
Risk of bias (quality) assessment	No	No
Data analysis	No	No

Provide any other relevant information about the stage of the review here.

6. * Named contact.

The named contact is the guarantor for the accuracy of the information in the register record. This may be any member of the review team.

KE YANI

Email salutation (e.g. "Dr Smith" or "Joanne") for correspondence:

Miss YANI

7. * Named contact email.

Give the electronic email address of the named contact.

2537086527@qq.com

8. Named contact address

Give the full institutional/organisational postal address for the named contact.

Zhejiang Chinese Medical University\nHangzhou ,Zhejiang

9. Named contact phone number.

Give the telephone number for the named contact, including international dialling code.

86-188-6713-3923

10. * Organisational affiliation of the review.

Full title of the organisational affiliations for this review and website address if available. This field may be

completed as 'None' if the review is not affiliated to any organisation.

Zhejiang Chinese Medical University

Organisation web address:

11. * Review team members and their organisational affiliations.

Give the personal details and the organisational affiliations of each member of the review team. Affiliation refers to groups or organisations to which review team members belong. **NOTE: email and country now MUST be entered for each person, unless you are amending a published record.**

Miss KE YANI. Zhejiang Chinese Medical University
Dr Yan Cai. Zhejiang Chinese Medical university
Dr Xing Bai. Zhejiang Chinese Medical University

12. * Funding sources/sponsors.

Details of the individuals, organizations, groups, companies or other legal entities who have funded or sponsored the review.

None

Grant number(s)

State the funder, grant or award number and the date of award

13. * Conflicts of interest.

List actual or perceived conflicts of interest (financial or academic).

None

14. Collaborators.

Give the name and affiliation of any individuals or organisations who are working on the review but who are not listed as review team members. **NOTE: email and country must be completed for each person, unless you are amending a published record.**

15. * Review question.

State the review question(s) clearly and precisely. It may be appropriate to break very broad questions down into a series of related more specific questions. Questions may be framed or refined using PI(E)COS or similar where relevant.

What is the relationship between Adipokine and HCC and what role does it play in disease progression?

16. * Searches.

State the sources that will be searched (e.g. Medline). Give the search dates, and any restrictions (e.g. language or publication date). Do NOT enter the full search strategy (it may be provided as a link or

attachment below.)

Database: PubMed, EMBASE, Cochrane Library, ClinicalTrials.gov, CNKI, WANFANG, CBM and grey literature

Publication period: up to December, 2023

Language: English and Chinese

Search strategy: (“hepatocellular carcinoma” OR “hepatocarcinoma” OR “primary liver cancer” OR “HCC” OR “hepatic carcinoma or liver tumor” OR “liver cancer” OR “liver tumor” OR “liver tumour” OR “liver malignance” OR “liver carcinoma” OR “liver neoplasm” OR “HCC-CC” OR “liver cell carcinoma” OR “hepatic cell carcinoma” OR “hepatoma”) AND (“adipokine” OR “adiponectin” OR “adiponectins” OR “adiponectine” OR “AdipoQ” OR “ACRP30” OR “Obese Protein” OR “Obese Gene Product” OR “ADPN” OR “APN” OR “apelin” OR “APLN” OR “APLNR” OR “apelin receptor” OR “AGTRL1 ligand, human” OR “Chemerin” OR “chemerin protein” OR “human TIG2 protein” OR “tazarotene induced gene?2 protein” OR “retinoic acid receptor responder protein 2” OR “RAR?responsive protein” OR “RARRES2 protein, human” OR “tazarotene-induced gene 2 protein” OR “TIG2” OR “leptin” OR “LEP” OR “adipocytokine” OR “leptin-binding protein” OR “leptin receptor” OR “ob protein” OR “ob gene product” OR “obesity factor” OR “Resistin” OR “RETN” OR “RELM” OR “FIZZ” OR “Adipose tissue-specific secretory factor” OR “XCP1” OR “C/EBP-epsilon-regulated myeloid-specific secreted cysteine-rich protein” OR “adipocyte secreted factor” OR “adipocyte specific secreted factor” OR “adipose tissue specific secreted factor” OR “Visfatin” OR “Nicotinamide Phosphoribosyltransferase” OR “NAMPT Protein” OR “Pre-B-Cell Colony-Enhancing Factor” OR “Pre B Cell Colony Enhancing Factor” OR “NAmPRTase” OR “NMN Pyrophosphorylase” OR “adipocyte specific secreted factor” OR “adipose tissue specific secreted factor” OR “Colony-Enhancing Factor, Pre-B-Cell” OR “PBEF” OR “irisin” OR “FNDC5” OR “fibronectin type III domain containing protein 5” OR “Fndc5 protein” OR “FRCP2 protein”)

17. URL to search strategy.

Upload a file with your search strategy, or an example of a search strategy for a specific database, (including the keywords) in pdf or word format. In doing so you are consenting to the file being made publicly accessible. Or provide a URL or link to the strategy. Do NOT provide links to your search **results**.

Alternatively, upload your search strategy to CRD in pdf format. Please note that by doing so you are consenting to the file being made publicly accessible.

Do not make this file publicly available until the review is complete

18. * Condition or domain being studied.

Give a short description of the disease, condition or healthcare domain being studied in your systematic review.

Obesity often leads to a series of medical disorders, including metabolic syndrome and nonalcoholic fatty liver disease (NAFLD), a spectrum including nonalcoholic steatohepatitis (NASH). It has also been recognized epidemiologically and clinically as a major risk factor for liver cancer. Because of its wide spread and high prevalence, obesity serves as a large contributor to overall hepatocellular carcinoma (HCC). The
precise mechanism underlying the obesity–liver cancer link are not yet completely understood
hepatic cells are mostly affected by obesity–lipid accumulation since the liver is the master regulator of systemic lipid and glucose accumulation. Consequently, NAFLD and NASH are the most common liver disorders, with up to a 90% prevalence in the obese population. They are strongly associated with insulin resistance, progressing to cirrhosis and eventually to HCC, highlighting that excess lipid deposition in the liver leads to severe pathological consequences. In recent years, there are more researches on adipokines and HCC. People gradually found that adipokine may play an important role in the process of diseases. We also found that many studies have explored the relationship between them. The results of these studies are not exactly the same, so we intend to analyse these data to draw more accurate conclusions.

19. * Participants/population.

Specify the participants or populations being studied in the review. The preferred format includes details of both inclusion and exclusion criteria.

Inclusion criteria: adults (aged 18 years or older); patients must be diagnosed with HCC; articles that focused on adipokine (including adiponectin, apelin, chemerin, leptin, resistin, visfatin and irisin).

20. * Intervention(s), exposure(s).

Give full and clear descriptions or definitions of the interventions or the exposures to be reviewed. The preferred format includes details of both inclusion and exclusion criteria.

Individuals with HCC, different stages of HCC, adipokine (including adiponectin, apelin, chemerin, leptin, resistin, visfatin and irisin) levels whether in plasma, serum or liver

21. * Comparator(s)/control.

Where relevant, give details of the alternatives against which the intervention/exposure will be compared (e.g. another intervention or a non-exposed control group). The preferred format includes details of both inclusion and exclusion criteria.

Healthy people without HCC or patients with different stages of HCC

22. * Types of study to be included.

Give details of the study designs (e.g. RCT) that are eligible for inclusion in the review. The preferred format includes both inclusion and exclusion criteria. If there are no restrictions on the types of study, this should be stated.

Case-control studies or cohort studies or cross-sectional studies

23. Context.

Give summary details of the setting or other relevant characteristics, which help define the inclusion or exclusion criteria.

24. * Main outcome(s).

Give the pre-specified main (most important) outcomes of the review, including details of how the outcome is defined and measured and when these measurement are made, if these are part of the review inclusion criteria.

the relationship between adipokine (including adiponectin, apelin, chemerin, leptin, resistin, visfatin and irisin) levels and patients with HCC

Measures of effect

Please specify the effect measure(s) for you main outcome(s) e.g. relative risks, odds ratios, risk difference, and/or 'number needed to treat.

25. * Additional outcome(s).

List the pre-specified additional outcomes of the review, with a similar level of detail to that required for main outcomes. Where there are no additional outcomes please state ‘None’ or ‘Not applicable’ as appropriate to the review

the relationship between adipokine levels and severity of HCC patients prognosis in patients with HCC

Measures of effect

Please specify the effect measure(s) for you additional outcome(s) e.g. relative risks, odds ratios, risk difference, and/or 'number needed to treat.

26. * Data extraction (selection and coding).

Describe how studies will be selected for inclusion. State what data will be extracted or obtained. State how this will be done and recorded.

Two members of the team are responsible for extracting information and data. The main information includes the first author's name, publication time, original country, NOS score, number of patients in each group, basic information of each group (age, sex and so on), measured method, diagnostic method, adipokine levels in each group, prognosis of each group. If there are any questions, they will be reviewed by a third member. If there is incomplete information, we will contact the author team of the article to obtain it.

27. * Risk of bias (quality) assessment.

State which characteristics of the studies will be assessed and/or any formal risk of bias/quality assessment tools that will be used.

All quality assessment is conducted using the NOS scale and independently by two members. The parts in question are left to the third member for decision. The NOS scale is commonly used for quality assessment in systematic reviews, which mainly includes several aspects: selectivity, comparability and exposure of each literature. The publication bias is based on Egger’s test and funnel plots. A p-value greater than 0.05 in Egger analysis is considered to have no significant publication bias. The relative symmetry of funnel plots is also considered to have no significant publication bias.

28. * Strategy for data synthesis.

Describe the methods you plan to use to synthesise data. This **must not be generic text** but should be **specific to your review** and describe how the proposed approach will be applied to your data. If meta-analysis is planned, describe the models to be used, methods to explore statistical heterogeneity, and software package to be used.

We will use a combination of Review Manager 5.3 and Stata 16 software. The heterogeneity among studies is determined by the p-value and I^2. When the heterogeneity among studies is relatively low, the fixed-effect model is the best choice. If the heterogeneity among studies is high, we will use the random-effect model. In addition, we will conduct subgroup analysis and meta-regression to further explore the possible sources of heterogeneity.

29. * Analysis of subgroups or subsets.

State any planned investigation of ‘subgroups’. Be clear and specific about which type of study or participant will be included in each group or covariate investigated. State the planned analytic approach.

Subgroups classified by different stages of HCC or by different ethnicities or by different treatment.

30. * Type and method of review.

Select the type of review, review method and health area from the lists below.

Type of review

Cost effectiveness

No

Diagnostic

No

Epidemiologic

No

Individual patient data (IPD) meta-analysis

No

Intervention

No

Living systematic review

No

Meta-analysis
Yes

Methodology
No

Narrative synthesis
No

Network meta-analysis
No

Pre-clinical
No

Prevention
No

Prognostic
No

Prospective meta-analysis (PMA)
No

Review of reviews
No

Service delivery
No

Synthesis of qualitative studies
No

Systematic review
Yes

Other
No

Health area of the review

Alcohol/substance misuse/abuse
No

Blood and immune system
No

Cancer
Yes

Cardiovascular
No

Care of the elderly

No

Child health

No

Complementary therapies

No

COVID-19

No

Crime and justice

No

Dental

No

Digestive system

No

Ear, nose and throat

No

Education

No

Endocrine and metabolic disorders

No

Eye disorders

No

General interest

No

Genetics

No

Health inequalities/health equity

No

Infections and infestations

No

International development

No

Mental health and behavioural conditions

No

Musculoskeletal

No

Neurological

No

Nursing

No

Obstetrics and gynaecology

No

Oral health

No

Palliative care

No

Perioperative care

No

Physiotherapy

No

Pregnancy and childbirth

No

Public health (including social determinants of health)

No

Rehabilitation

No

Respiratory disorders

No

Service delivery

No

Skin disorders

No

Social care

No

Surgery

No

Tropical Medicine

No

Urological

No

Wounds, injuries and accidents

No

Violence and abuse

No

31. Language.

Select each language individually to add it to the list below, use the bin icon to remove any added in error.

English

There is not an English language summary

32. * Country.

Select the country in which the review is being carried out. For multi-national collaborations select all the countries involved.

China

33. Other registration details.

Name any other organisation where the systematic review title or protocol is registered (e.g. Campbell, or The Joanna Briggs Institute) together with any unique identification number assigned by them. If extracted data will be stored and made available through a repository such as the Systematic Review Data Repository (SRDR), details and a link should be included here. If none, leave blank.

34. Reference and/or URL for published protocol.

If the protocol for this review is published provide details (authors, title and journal details, preferably in Vancouver format)

Add web link to the published protocol.

Or, upload your published protocol here in pdf format. Note that the upload will be publicly accessible.

No I do not make this file publicly available until the review is complete

Please note that the information required in the PROSPERO registration form must be completed in full even if access to a protocol is given.

35. Dissemination plans.

Do you intend to publish the review on completion?

No

Give brief details of plans for communicating review findings.?

36. Keywords.

Give words or phrases that best describe the review. Separate keywords with a semicolon or new line. Keywords help PROSPERO users find your review (keywords do not appear in the public record but are included in searches). Be as specific and precise as possible. Avoid acronyms and abbreviations unless these are in wide use.

37. Details of any existing review of the same topic by the same authors.

If you are registering an update of an existing review give details of the earlier versions and include a full bibliographic reference, if available.

38. * Current review status.

Update review status when the review is completed and when it is published.New registrations must be ongoing so this field is not editable for initial submission.

Please provide anticipated publication date

Review_Ongoing

39. Any additional information.

Provide any other information relevant to the registration of this review.

40. Details of final report/publication(s) or preprints if available.

Leave empty until publication details are available OR you have a link to a preprint (NOTE: this field is not editable for initial submission). List authors, title and journal details preferably in Vancouver format.

Give the link to the published review or preprint.
