# Supplementary material for: Correlations between circulating adipokines and hepatocellular carcinoma: a Systematic Review and meta-analysis
Source: Front Endocrinol (Lausanne). 2025 Jun 18;16:1548924. doi: 10.3389/fendo.2025.1548924 (PMC12216435; doi:10.3389/fendo.2025.1548924)
Supplement: Supplementary Data Sheet 3 — Databases retrieval strategy. [file DataSheet3.docx]

| Databases | Search terms | Number of records |
| --- | --- | --- |
| Pubmed | Search：(('hepatocellular carcinoma'[Title/Abstract])) OR ('hepatocarcinoma'[Title/Abstract]) OR ('primary liver cancer'[Title/Abstract]) OR ('HCC'[Title/Abstract]) OR ('hepatic carcinoma or liver tumor'[Title/Abstract]) OR ('liver cancer'[Title/Abstract]) OR ('liver tumor'[Title/Abstract]) OR ('liver tumour'[Title/Abstract]) OR ('liver malignance'[Title/Abstract]) OR ('liver carcinoma'[Title/Abstract]) OR ('liver neoplasm'[Title/Abstract]) OR ('HCC-CC'[Title/Abstract]) OR ('liver cell carcinoma'[Title/Abstract]) OR ('hepatic cell carcinoma'[Title/Abstract]) OR ('hepatoma'[Title/Abstract]) OR ('hepatocellular carcinoma'[MeSH Terms])) AND (('adipokine') OR ('adiponectin') OR ('adiponectins') OR ('adiponectine') OR ('AdipoQ') OR ('ACRP30') OR ('Obese Protein') OR ('Obese Gene Product') OR ('ADPN') OR ('APN') OR ('adiponectin'[MeSH Terms]) OR ('apelin') OR ('APLN') OR ('APLNR') OR ('apelin receptor') OR ('AGTRL1 ligand, human') OR ('apelin'[MeSH Terms]) OR ('Chemerin') OR ('chemerin protein') OR ('human TIG2 protein') OR ('tazarotene induced gene‐2 protein') OR ('retinoic acid receptor responder protein 2') OR ('RAR‐responsive protein') OR ('RARRES2 protein, human') OR ('tazarotene-induced gene 2 protein') OR ('TIG2') OR ('Chemerin'[MeSH Terms]) OR ('leptin') OR ('LEP') OR ('adipocytokine') OR ('leptin-binding protein') OR ('leptin receptor') OR ('ob protein') OR ('ob gene product') OR ('obesity factor') OR ('leptin'[MeSH Terms]) OR ('Resistin') OR ('RETN') OR ('RELM') OR ('FIZZ') OR ('Adipose tissue-specific secretory factor') OR ('XCP1') OR ('C/EBP-epsilon-regulated myeloid-specific secreted cysteine-rich protein') OR ('adipocyte secreted factor') OR ('adipocyte specific secreted factor') OR ('adipose tissue specific secreted factor') OR ('Resistin'[MeSH Terms]) OR ('Visfatin') OR ('Nicotinamide Phosphoribosyltransferase') OR ('NAMPT Protein') OR ('Pre-B-Cell Colony-Enhancing Factor') OR ('Pre B Cell Colony Enhancing Factor') OR ('NAmPRTase') OR ('NMN Pyrophosphorylase') OR ('Colony-Enhancing Factor, Pre-B-Cell') OR ('PBEF') OR ('Visfatin'[MeSH Terms]) OR ('irisin') OR ('FNDC5') OR ('fibronectin type III domain containing protein 5') OR ('Fndc5 protein') OR ('FRCP2 protein') OR ('irisin'[MeSH Terms])) | 2702 |
| Cochrane Library | #1MeSH descriptor:[adipokine] explode all trees  #2MeSH descriptor:[adiponectin] explode all trees  #3MeSH descriptor:[apelin] explode all trees  #4MeSH descriptor:[leptin] explode all trees  #5MeSH descriptor:[Resistin] explode all trees  #6MeSH descriptor:[Visfatin] explode all trees  #7('adiponectin' OR 'adiponectins' OR 'adipokine' OR 'AdipoQ' OR 'ACRP30' OR 'Obese Protein' OR 'Obese Gene Product' OR 'ADPN' OR 'APN' OR 'apelin' OR 'APLN' OR 'APLNR' OR 'apelin receptor' OR 'AGTRL1 ligand, human' OR 'Chemerin' OR 'chemerin protein' OR 'human TIG2 protein' OR 'tazarotene induced gene‐2 protein' OR 'retinoic acid receptor responder protein 2' OR 'RAR‐responsive protein' OR 'RARRES2 protein, human' OR 'tazarotene-induced gene 2 protein' OR 'TIG2' OR 'leptin' OR 'LEP' OR 'adipocytokine' OR 'leptin-binding protein' OR 'leptin receptor' OR 'ob protein' OR 'ob gene product' OR 'obesity factor' OR 'Resistin' OR 'RETN' OR 'RELM' OR 'FIZZ' OR 'Adipose tissue-specific secretory factor' OR 'XCP1' OR 'CEBP-epsilon-regulated myeloid-specific secreted cysteine-rich protein' OR 'adipocyte secreted factor' OR 'adipocyte specific secreted factor' OR 'adipose tissue specific secreted factor' OR 'Visfatin' OR 'Nicotinamide Phosphoribosyltransferase' OR 'NAMPT Protein' OR 'Pre B Cell Colony Enhancing Factor' OR 'NMN Pyrophosphorylase' OR 'Colony-Enhancing Factor, Pre-B-Cell' OR 'PBEF' OR 'irisin' OR 'FNDC5' OR 'fibronectin type III domain containing protein 5' OR 'Fndc5 protein' OR 'FRCP2 protein'):ti,ab,kw  #8#1 or #2 or #3 or #4 or #5 or #6 or #7  #9('hepatocellular carcinoma' OR 'hepatocarcinoma' OR 'primary liver cancer' OR 'hcc' OR 'hepatic carcinoma or liver tumor' OR 'liver cancer' OR 'liver tumor' OR 'liver tumour' OR 'liver carcinoma' OR 'liver neoplasm' OR 'hcc-cc' OR 'liver cell carcinoma' OR 'hepatic cell carcinoma' OR 'hepatoma') :ti,ab,kw  #10MeSH descriptor:[hepatocellular carcinoma] explode all trees  #11#9 OR #10  #12#8 AND #11 | 455 |
| EMBASE | #1'adipokine'/exp OR 'adipokine' OR 'adiponectin'/exp OR 'adiponectin' OR 'adiponectin':ti,ab,kw OR 'adiponectins':ti,ab,kw OR 'adipokine':ti,ab,kw OR 'adipoq':ti,ab,kw OR 'acrp30':ti,ab,kw OR 'obese protein':ti,ab,kw OR 'obese gene product':ti,ab,kw OR 'adpn':ti,ab,kw OR 'apn':ti,ab,kw OR 'apelin':ti,ab,kw OR 'apln':ti,ab,kw OR 'aplnr':ti,ab,kw OR 'apelin receptor':ti,ab,kw OR 'agtrl1 ligand, human':ti,ab,kw OR 'apelin'/exp OR 'apelin' OR 'chemerin':ti,ab,kw OR 'chemerin protein':ti,ab,kw OR 'human tig2 protein':ti,ab,kw OR 'tazarotene induced gene‐2 protein':ti,ab,kw OR 'retinoic acid receptor responder protein 2':ti,ab,kw OR 'rar‐responsive protein':ti,ab,kw OR 'rarres2 protein, human':ti,ab,kw OR 'tazarotene-induced gene 2 protein':ti,ab,kw OR 'tig2':ti,ab,kw OR 'chemerin'/exp OR 'chemerin' OR 'leptin':ti,ab,kw OR 'lep':ti,ab,kw OR 'adipocytokine':ti,ab,kw OR 'leptin-binding protein':ti,ab,kw OR 'leptin receptor':ti,ab,kw OR 'ob protein':ti,ab,kw OR 'ob gene product':ti,ab,kw OR 'obesity factor':ti,ab,kw OR 'leptin'/exp OR 'leptin' OR 'resistin':ti,ab,kw OR 'retn':ti,ab,kw OR 'relm':ti,ab,kw OR 'fizz':ti,ab,kw OR 'adipose tissue-specific secretory factor':ti,ab,kw OR 'xcp1':ti,ab,kw OR 'c/ebp-epsilon-regulated myeloid-specific secreted cysteine-rich protein':ti,ab,kw OR 'adipocyte secreted factor':ti,ab,kw OR 'adipocyte specific secreted factor':ti,ab,kw OR 'adipose tissue specific secreted factor':ti,ab,kw OR 'resistin'/exp OR 'resistin' OR 'visfatin':ti,ab,kw OR 'nicotinamide phosphoribosyltransferase':ti,ab,kw OR 'nampt protein':ti,ab,kw OR 'pre b cell colony enhancing factor':ti,ab,kw OR 'nmn pyrophosphorylase':ti,ab,kw OR 'colony-enhancing factor, pre-b-cell':ti,ab,kw OR 'pbef':ti,ab,kw OR 'visfatin'/exp OR 'visfatin' OR 'irisin':ti,ab,kw OR 'fndc5':ti,ab,kw OR 'fibronectin type iii domain containing protein 5':ti,ab,kw OR 'fndc5 protein':ti,ab,kw OR 'frcp2 protein':ti,ab,kw OR 'irisin'/exp OR 'irisin':ti,ab,kw  #2 'hepatocellular carcinoma'/exp OR 'hepatocellular carcinoma':ti,ab,kw OR 'hepatocarcinoma':ti,ab,kw OR 'primary liver cancer':ti,ab,kw OR 'HCC':ti,ab,kw OR 'hepatic carcinoma or liver tumor':ti,ab,kw OR 'liver cancer':ti,ab,kw OR 'liver tumor':ti,ab,kw OR 'liver tumour':ti,ab,kw OR 'liver carcinoma':ti,ab,kw OR 'liver neoplasm':ti,ab,kw OR 'HCC-CC':ti,ab,kw OR 'liver cell carcinoma':ti,ab,kw OR 'hepatic cell carcinoma':ti,ab,kw OR 'hepatoma':ti,ab,kw  #3#1 AND #2 | 1489 |
| CNKI | (SU='肝脏肿瘤' OR SU='肝部肿瘤' OR SU='肝癌' OR SU='原发性肝癌' OR SU='肝恶性肿瘤' OR SU='原发性肝细胞癌' OR SU='肝脏恶性肿瘤' OR SU='肝细胞癌' OR SU='肝肿瘤' OR SU='HCC') AND (SU='脂肪因子' OR SU='脂联素' OR SU='adiponectin' OR SU='艾帕素' OR SU='爱帕琳肽' OR SU='G蛋白耦联受体配体' OR SU='apelin' OR SU='趋化素' OR SU='Chemerin' OR SU='瘦素' OR SU='leptin' OR SU='抵抗素' OR SU='Resistin' OR SU='内脏脂肪素' OR SU='内脂素' OR SU='Visfatin' OR SU='鸢尾素' OR SU='irisin') | 139 |
| Wanfang | 提名或关键词：(("肝脏肿瘤" or "肝部肿瘤" or "肝癌" or "原发性肝癌" or "肝恶性肿瘤" or "原发性肝细胞癌" or "肝脏恶性肿瘤" or "肝细胞癌" or "肝肿瘤" or "HCC") and ("脂肪因子" or "脂联素" or "adiponectin" or "艾帕素" or "爱帕琳肽" or "G蛋白耦联受体配体" or "apelin" or "趋化素" or "Chemerin" or "瘦素" or "leptin" or "抵抗素" or "Resistin" or "内脏脂肪素" or "内脂素" or "Visfatin" or "鸢尾素" or "irisin")) | 100 |
| CBM | ("脂肪因子"[全部字段] OR "脂联素"[全部字段] OR "adiponectin"[全部字段] OR "艾帕素"[全部字段] OR "爱帕琳肽" OR "G蛋白耦联受体配体"[全部字段] OR "apelin"[全部字段] OR "趋化素"[全部字段] OR "Chemerin"[全部字段] OR "瘦素"[全部字段] OR "leptin"[全部字段] OR " 抵抗素"[全部字段] OR "Resistin"[全部字段] OR "内脏脂肪素"[全部字段] OR "内脂素"[全部字段] OR "Visfatin"[全部字段] OR "鸢尾素"[全部字段] OR "irisin"[全部字段]) AND ("肝脏肿瘤"[全部字段] OR "肝部肿瘤"[全部字段] OR "肝癌"[全部字段] OR "原发性肝癌"[全部字段] OR "肝恶性肿瘤"[全部字段] OR "原发性肝细胞癌"[全部字段] OR "肝脏恶性肿瘤"[全部字段] OR "肝细胞癌"[全部字段] OR "肝肿瘤"[全部字段] OR "HCC"[全部字段]) | 154 |
